# Re-operation beating heart valve surgery post myocardial revascularization with patent grafts

**DOI:** 10.1186/1749-8090-8-S1-O5

**Published:** 2013-09-11

**Authors:** P Pepino, G Coronella, P Oliviero, A Contaldo, R Provenzano, S Giordano, V Pota, A Luciano, M Monaco, V Schiavone

**Affiliations:** 1 Department of Cardiothoracic Surgery, Presidio Ospedaliero Pineta Grande, Castel Volturno, Italy; 2 Department of Anaesthesiology and Intensive Care, Castel Volturno, Italy

## Background

Valve surgery in patients with previous CABG and patent arterials conducts is a challenging procedure. To avoid any risk of injury the grafts, we performed valve surgery with the beating heart: 4 aortic valve replacement (AVR); 1 ascending aorta replacement and 2 mitral valve replacement (MVR).

## Methods

From September 2010 up to May 2013 seven patients (four male and three female, aged 67,66±10,85, pre op LVEF 47,85±6,46) with a story of previous CABG (three patients using left internal mammary artery + saphenous vein; four patients using LIMA + radial artery; one patient using also the right internal mammary artery, number of by pass 2.85±0.63) were admitted in our department for valvular or ascending aorta disease. All patients had previous coronarography with patent arterial conduit in six patients, while in one patient an occluded LIMA graft on the LAD and a patent radial artery graft from the LIMA on the first and second obtuse marginal were observed. Reoperations were performed on beating heart. Arterial conducts were not isolated to avoid possible injury and the procedures were performed cross-clamping the aorta, on pump, perfusing the coronary trough the patents graft and with a retrograde perfusion with a cannula in the coronary sinus.

## Results

The aortic cross clamp time was 81,8±20,1. Two patients had AVR with biological valve, two patients had AVR with mechanical valve, one patient had replacement of the ascending aorta with a dacron graft and two patients had MVR with biologic valve. No major complications were observed. No in hospital mortality was observed and all patients were discharged in 6,5±0,9 days post op, showing a LVEF of 49,28±5,62.

## Conclusion

Beating heart surgery is a safe procedure in patients who received a previous CABG. Maintaining coronary perfusion through the patents arterial conducts and through the retrograde perfusion will protect the heart without any ischemia.

